# Hybrid sequencing and map finding (HySeMaFi): optional strategies for extensively deciphering gene splicing and expression in organisms without reference genome

**DOI:** 10.1038/srep43793

**Published:** 2017-03-08

**Authors:** Guogui Ning, Xu Cheng, Ping Luo, Fan Liang, Zhen Wang, Guoliang Yu, Xin Li, Depeng Wang, Manzhu Bao

**Affiliations:** 1 Key laboratory of Horticultural Plant Biology, Ministry of Education, College of Horticulture and Forestry Sciences, Huazhong Agricultural University, Wuhan, P. R. China; 2 Medical Research Institute, School of Medicine, Wuhan University, Wuhan, P. R. China; 3 Nextomics Biosciences Co., Ltd., Wuhan, Hubei, China

## Abstract

Using second-generation sequencing (SGS) RNA-Seq strategies, extensive alterative splicing prediction is impractical and high variability of isoforms expression quantification is inevitable in organisms without true reference dataset. we report the development of a novel analysis method, termed hybrid sequencing and map finding (HySeMaFi) which combines the specific strengths of third-generation sequencing (TGS) (PacBio SMRT sequencing) and SGS (Illumina Hi-Seq/MiSeq sequencing) to effectively decipher gene splicing and to reliably estimate the isoforms abundance. Error-corrected long reads from TGS are capable of capturing full length transcripts or as large partial transcript fragments. Both true and false isoforms, from a particular gene, as well as that containing all possible exons, could be generated by employing different assembly methods in SGS. We first develop an effective method which can establish the mapping relationship between the error-corrected long reads and the longest assembled contig in every corresponding gene. According to the mapping data, the true splicing pattern of the genes was reliably detected, and quantification of the isoforms was also effectively determined. HySeMaFi is also the optimal strategy by which to decipher the full exon expression of a specific gene when the longest mapped contigs were chosen as the reference set.

Short-read second-generation sequencing (SGS) has become a powerful tool for the description of gene expression levels and individual splice junctions in those organisms with a reference genome^1^,^2^,^3^. Despite the availability of verified and improved algorithms along with good software options, it is still difficult to identify full-length transcript isoforms using the SGS data. Thus, it is not yet possible to get a complete understanding of all spliced RNAs within a transcriptome using SGS, even in those organisms with a reference genome^4^. Given that, even with a true reference sequence, spliced RNAs can only be partly inferred from a patchwork of short fragments, it is not surprising that, to date, there are no reports of the successful performance of gene splicing analyses by SGS in organisms lacking a reference genome. At the same time, it is well recognized that reconstruction and quantification of transcript isoforms from short-read sequencing is insufficiently accurate^5^,^6^.

Transcriptional and alternative splicing events may be detected, to some extent, in RNA-Seq data from SGS. However, full-length mRNA isoforms are not directly captured or may even not be detected, despite the employment of various powerful computational algorithms and software. Pacific Biosciences (PacBio) developed a novel platform enabling single molecule real time (SMRT) sequencing^7^, and this represented an advance over SGS, i.e. third-generation sequencing (TGS). TGS, although currently having the limitations of reduced raw accuracy and lower throughput, has significant capacity for application in *de novo* sequencing, and may also aid the analysis of linkage of alternative splicing forms and of variants across long amplicons, as has been successfully used in the study of the human genome and of other organisms with an available reference genome^4^. In the past, several approaches have been developed that utilize short high-identity sequences to correct the error inherent in long single-molecule sequences, and thereby generate a highly accurate hybrid consensus sequence^8^,^9^. With the development of improved correction of base-call errors in TGS, the technique is increasingly widely used in genome sequencing due to its advantage of generating long reads^10^,^11^,^12^. To date, the advantages of SMRT sequencing have been utilized in order to identify extensive alternative splicing patterns and also thousands of transcript isoforms from numerous organisms which have a known reference genome^13^. However, no strategy or methodology currently exists that can extensively decipher gene splicing or efficiently calculate the expression of alternative isoforms on a large scale in an organism for which the genome sequence is not available.

Many eukaryotic genes exhibit extensive alternative splicing, and alternative splicing (AS) is of importance as it represents a posttranscriptional mechanism that can mediate regulation of gene expression^14^,^15^. The high rate of occurrence of AS in plants during different developmental stages or when growing under diverse environmental conditions has sparked a growing interest in AS as a regulatory mechanism, and has led to further studies focused on revealing the full extent of AS by deep sequencing^16^,^17^. It is our understanding that, using the analysis techniques currently available, it is not possible to routinely and accurately decipher the AS profile based solely on SGS if a detailed genome annotation is not available. SGS is capable of generating a large number of short reads to support the databases produced by current RNA-Seq technology. Based on short reads, the following information can be obtained: (i) frequency of reads mapped to a contiguous genomic segment (exonic reads), and (ii) frequency of reads mapped to two contiguous segments of the genome with a single gap (junction reads)^18^,^19^,^20^. It is possible to infer isoform-specific expression from exonic reads and junction reads if the full set of possible isoforms for the given gene is already established. However, such isoform quantification presents significant difficulties if the set of isoforms is not known or only partially known due to insufficient lengths of the SGS reads^21^. Thus, taking mentions above, it is possible to deduce a methodology that could extensively decipher gene splicing and effectively compute gene expression profiles using close-to-real levels of isoforms.

In this paper, we initially extracted four distinct tissue types in order to generate extensive data sets using TGS, SGS and MiSeq methods. We used these datasets to develop a novel analysis technique which is based on the ability of error-corrected long reads in TGS to capture many transcripts in full length or, at least, as large partial transcript fragments. Furthermore, the various alternative contigs, including the true and false forms and also that containing the full complement of exons of a particular gene, can be generated by employing the different suitable assembly methods in SGS. Based on these data outputs, we have developed an effective mapping method, coupled with an associated analysis pipeline, which we demonstrate is able to establish the mapping relationship between the error-corrected long reads and the longest assembled contig in every corresponding gene. The mapping method was also tested and verified using the existing annotated isoform sets and the contigs datasets assembled from SGS by *de novo* assembly in *Arabidopsis*. Secondly, relying on the mapping technique, the longest contigs, typically comprising the full complement of exons corresponding to individual genes, were selected to form a reference library, and then from this, extensive gene splicing patterns were determined. The alternative splicing sites were verified by datasets from MiSeq. Thirdly, by utilizing the corrected long reads of isoforms in TGS or the mapped longest contigs from SGS as reference materials, and combining the alignments with a large number of short reads from different samples, the expression quantification of isoforms of genes was also effectively determined. In summary, a novel optional strategy, coupled with its analysis pipeline, were developed and demonstrated, which allows efficient deciphering of gene splicing and isoform expression in organisms whose genome is unavailable; this method is described as hybrid sequencing and map finding (HySeMaFi).

## Results

### Principles of hybrid sequencing and map finding (HySeMaFi)

**Figure 1:**
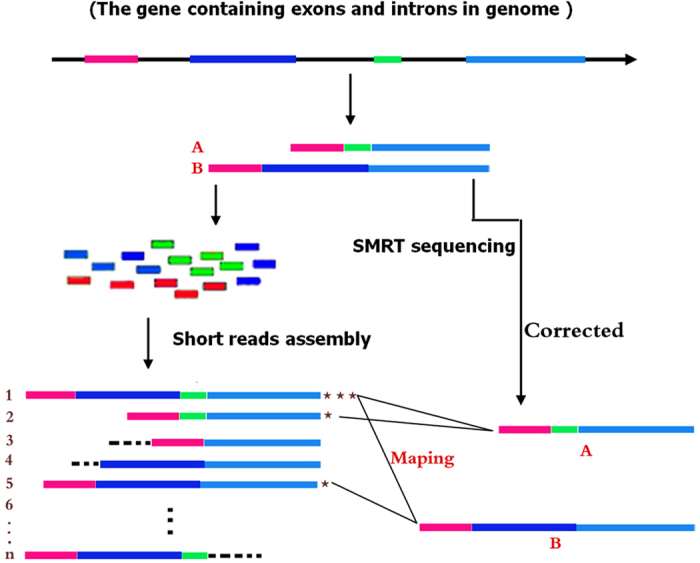
A general (theoretical) scheme for the hybrid sequencing and map finding (HySeMaFi) method. Based on each gene containing exons and introns, it is hypothesised that (**A,B**) isoforms are generated in particular tissues, and then two sequencing strategies are employed to yield the multiple molecules. In SGS, it is may be that all possible molecule forms are produced when employing different assembly methods with various parameters, wherein the real (true) molecule is contained. In comparison, in single molecule real-time (SMRT) sequencing (TGS), only two tissue-specific real molecules (after correction) were generated. It is possible to map the molecules between SGS and TGS molecular forms using our developed mapping methods. Finally, the molecule containing all of the gene exons in SGS is detected and used to follow downstream analysis.

The basic principle of the HySeMaFi technique is outlined in Fig. 1 (and detailed in Supplementary Fig. S1). The method centers on the SGS and TGS technologies and the mapping between the single-molecule corrected PacBio reads and the assembled contigs derived from SGS. It is hypothesised that if the A and B isoforms from a given gene truly exist in specific cell, tissue or organ types, then the application of two sequencing strategies will generate two distinct transcript datasets (Fig. 1). Through the SGS and assembly technique, it is theoretically unavoidable that various different molecule sets, containing the true molecules and/or false ones, could be generated when employing different assembly computation methods with alternative parameters, such as using shorter K-mer and low coverage values (Fig. 1). Thus the longest molecule (i.e. including all exons of the gene) was assembled by combining all the other real, as well as false, molecules that were generated in a specific tissue type (Fig. 1 and Supplementary Fig. S2). If all genes were considered, then more alternative molecules were obtained and there was a high level of confidence that the assembled contig pool contained the longest molecule of every gene, as well as the other real isoforms of each (Fig. 1). In TGS, PacBio reads, after correction for short reads or self-alignments, represent full-length or near full-length true transcripts (Fig. 1 and Supplementary Fig. S2). Therefore, it is entirely feasible that we could establish the mapping relationship between the longest molecule in SGS and the corrected PacBio reads (Fig. 1 and Supplementary Fig. S2) using suitable alignment methods. Thus, taking the corrected PacBio long reads and the longest contig into consideration, this scheme shows great potential to extensively decipher gene splicing by reasonable alignment analysis (Fig. 1 and Supplementary Fig. S2). In addition, it is also apparent that, if the longest contigs from SGS or the corrected PacBio long reads from TGS are employed as reference input data, the expression abundance of the gene or of each isoform, specific to a given tissue, will be effectively calculated (Supplementary Fig. S1). Thus, patterns of gene splicing and expression may be effectively determined through this approach, even in those organisms without an available reference genome.

### Contigs derived from SGS

**Figure 2:**
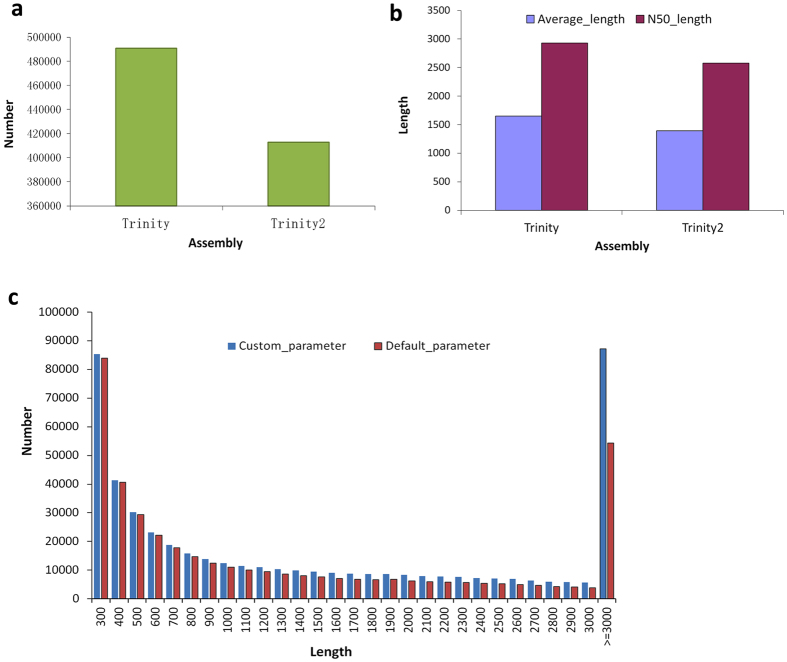
The statistics of assembly contigs derived from SGS. (**a**) The number of assembled contigs by Trinity assembler using low threshold or default parameters. (**b**) Average and N50 length of contigs under the two different assembly parameters. (**c**) Distribution of the number of contigs at various lengths.

The sequencing of 12 *Petunia hybrida* cDNA libraries, derived from roots, stems, leaves and flowers, generated a total of 945,501,540 clean reads (Accession number SRR4116645–56 at NCBI) after removal of adapter sequences and low quality reads, and each of the libraries yielded high quality reads in the range 63–84 M (Supplementary Table S1). To maximize transcript coverage, we pooled all of the clean Illumina reads together in order to perform *de novo* transcriptome assembly using the Trinity assembler employing various parameters. To obtain the maximum number of assembled theoretic nucleotide molecules, we employed either low threshold or default parameters in the assembly process and consequently produced two different *de novo* transcriptome versions containing 490,981 and 412,941 transcripts (genes), respectively (Fig. 2a and Supplementary Table S2). The statistics describing these assembled data sets are given in Table S2. The average contig length that was assembled using Trinity under low threshold parameters was 1648.15 bp and the N50 value was 2930 bp; this compares with the respective values of 1394.38 bp and 2579 bp when default parameters were used in the assembly process (Fig. 2b and Supplementary Table S2). Comparative analysis indicated that longer molecules (≥3000 bp) were produced when the custom parameters were employed (Fig. 2c). In MiSeq, more than 10 M high quality paired-end MiSeq reads were achieved (Supplementary Table S3). After processing, we obtained 4,596,458 extended fragments with an average length of 444 bp (Supplementary Table S4), and the distribution of extended fragments length ranged between 300–590 bp (Supplementary Fig. S3). In additional, clean reads after removal low quality reads in *Arabidopsis* were summarized as Table S5, and two different *de novo* transcriptome versions generate 44,934 and 44,914 genes (Supplementary Table S6).

### Long, corrected PacBio reads derived from TGS

**Figure 3:**
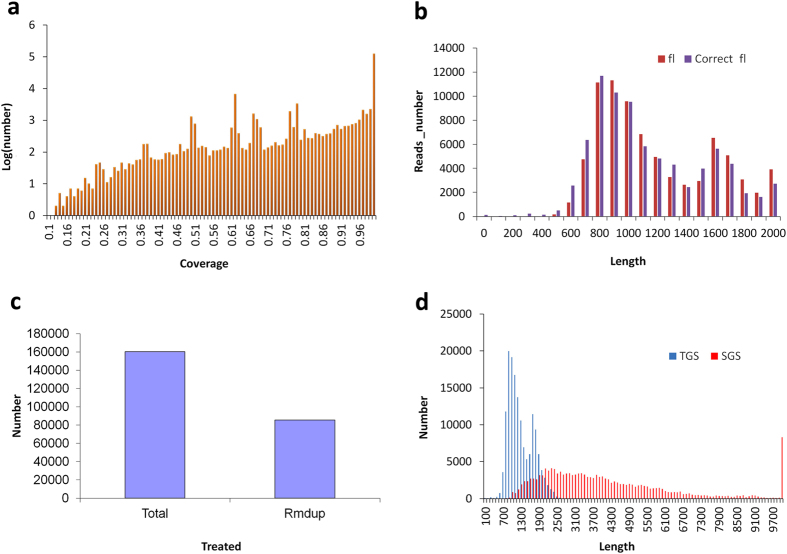
The statistics of PacBio long reads derived from TGS and corrected by LSC using Hiseq sequencing data. (**a**) PacBio corrected long reads supported by the Hiseq data with varied coverage. (**b**) Numbers distribution of PacBio long reads before and after correction using LSC. (**c**) Number of PacBio corrected long reads before and after duplication-removal using our developed methods. (**d**) The number distribution of PacBio corrected long reads and Hiseq assembled contigs at various lengths.

Based on the two SMRT sequence libraries, six SMRT cells were employed to generate the PacBio raw sequence data. The number of insert reads in each SMRT cell ranged from 46k to 54k (Supplementary Table S7), with an average of >90% quality. In total, after the classification analysis, we obtained 299,542 reads of inserts of which approximately 53.7% (i.e. 160,728) represented full-length reads (Accession number SRR4117145-46 at NCBI) after raw data processing (Supplementary Table S8). Moreover, the number distribution for insert length across the various reads indicates that more than 20 K insert-reads were obtained (Supplementary Fig. S4a) and most of these were of a high quality (Supplementary Fig. S4b). For the full-length non-chimeric reads, the distribution of read lengths was consistent with the level of transcript lengths seen in other plant species (Supplementary Fig. S5). In our study, we adapted 3 strategies in order to achieve the high quality PacBio-read dataset: namely, (1) full-length reads with an accuracy greater than 99% were isolated after removing reads shorter than 200 bp; (2) consensus isoforms were predicted using ICE software and then polished using Quiver software, after the removal of any reads shorter than 200 bp or with a predicted accuracy lower than 75%; (3) the full length transcripts were corrected by using LSC with SGS short reads. In the LSC correction process, most of the full length transcripts were supported by the SGS short reads, and a coverage value of more than 99% represented the highest number of short reads (Fig. 3a). After correction, very few differences were seen between the corrected PacBio reads derived from consensus information and the primary PacBio reads (Fig. 3b). At the end of the analysis process, we obtained a total of 160,293 error-corrected long reads. Duplicate long reads were removed by undertaking the clustering based on map finding according to our algorithm, and a total of 85,571 unique long reads were generated (Fig. 3c). A comparison to the transcripts assembled by SGS showed that the distribution of lengths of the corrected PacBio long reads appeared to more typically resemble the true transcript lengths (i.e. 1–3 Kb) seen in plants. This is significantly different to the transcriptome data assembled from SGS with regard to the frequency of long transcripts (Fig. 3d).

### Contigs Mapping between corrected PacBio reads and contigs from SGS

**Figure 4:**
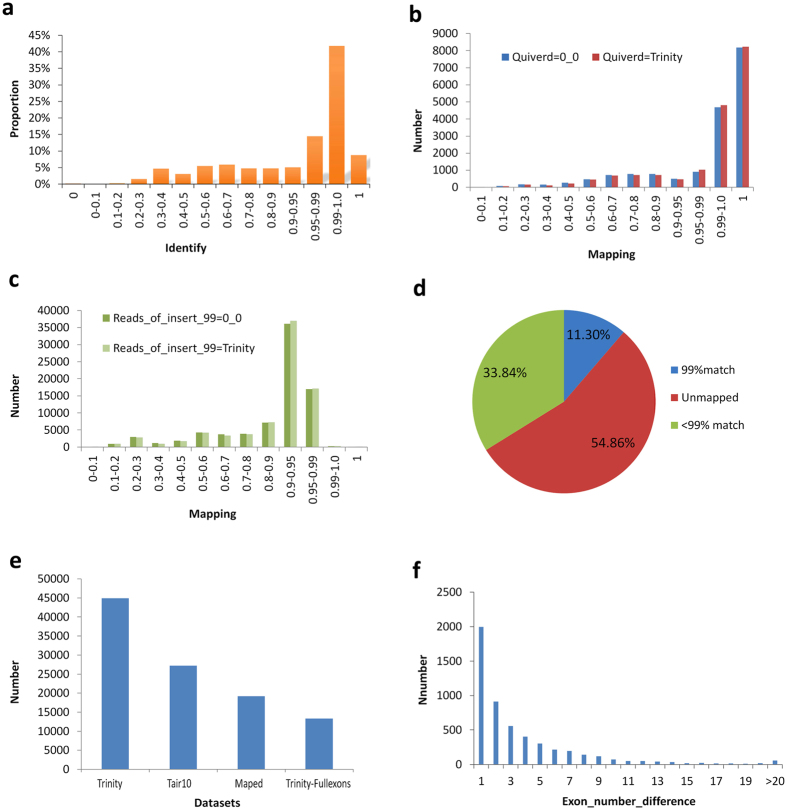
The statistics of mapping between PacBio corrected long reads of TGS and de novo assembled contigs of SGS. (**a**) The mapping between PacBio corrected long reads using LSC method and the assembled contigs using low threshold parameters at various identity rates. (**b**) The mapping between PacBio corrected long reads using ICE and Quiver software, and the assembled contigs using low threshold or default parameters. (**c**) The mapping between PacBio corrected long reads with a more than 99% corrected rate, and the assembled contigs using low threshold or default parameters. (**d**) Pie chart of mapped or unmapped analysis to the contigs as assembled in total from SGS by Trinity software with a 99% level mapping threshold. (**e**) The mapping between the existing annotated isoform sets and the contigs datasets assembled from SGS by *de novo* assembly in *Arabidopsis*. (**f**) The exon number difference between the existing annotated isoform sets and the contigs datasets assembled from SGS in *Arabidopsis* in many mapping.

Using the LSC corrected PacBio reads as the query sequences, the contigs from SGS (Illumina Hi-Seq) were aligned by Trinity using default parameters, and a total of 85,571 unique reads were mapped to specific transcripts according to our novel redundancy-removal and mapping method. Our process does not rely on generalized identifications based on gene structures and, therefore, is relatively unbiased. Of these mapped PacBio reads, 50% (43,132) had a more than 99% identity rate (Fig. 4a). To test our novel analysis method, a further two corrected data sets derived from TGS were employed to perform the mapping analysis. Using the consensus isoforms predicted using ICE software and then polished by Quiver as the query sequences, it was observed that over 13 thousand, or 12 thousand of the PacBio long reads could be mapped to the transcripts assembled using Trinity when employing default, or low stringency parameters, respectively, at the more than 99% identify threshold (Fig. 4b). However, using the remaining full-length reads with an accuracy level greater than 99% as defined by TGS sequencing, few of the long reads could be mapped to the Trinity-assembled transcripts (Fig. 4c). It is clear that the higher error rate in SMRT sequencing persists despite the removal of low quality data. In comparison, of the approx. 491,000 transcripts assembled in SGS, more than 33% of transcripts could not be mapped to the PacBio corrected long reads, and only 55,000 transcripts (11.30%) could be mapped to the PacBio corrected long reads with a 99% identity level (Fig. 4d). This indicates that a large number of untrue transcripts are unavoidably generated as part of the SGS assembly process. In *Arabidopsis*, total existing 19194 annotated isoforms could map to specific transcripts from SGS by *de novo* assembly just using three downloaded datasets according to our novel redundancy-removal and mapping method, of which total 13336 transcripts contains full exons (Fig. 4e). In those non-mapping genes, it exists many differences in exon number (Fig. 4f).

### Gene alternative splicing detection

**Figure 5:**
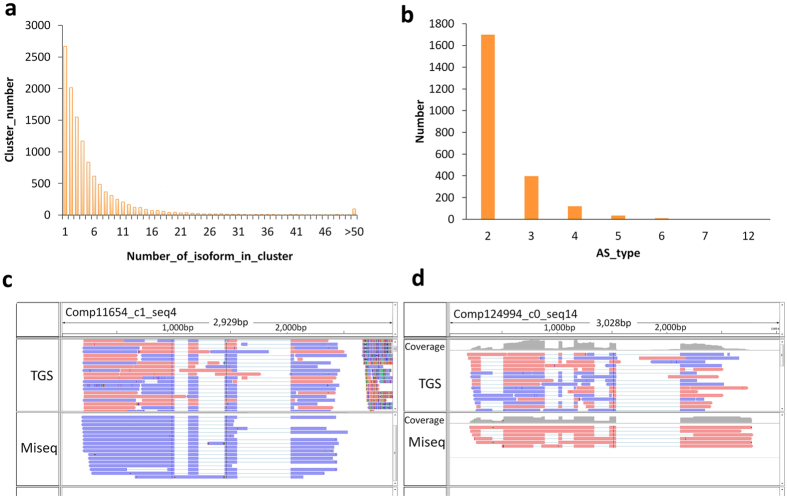
Extensive identification of gene alternative splicing patterns at a global level using HySeMaFi method. (**a**) Clustering of the SGS assembled contigs (genes) mapped by the PacBio corrected long reads. (**b**) The statistics of the gene numbers with various alternative splicing forms. (**c,d**) Cases selected to verify the effectiveness of detecting the isoforms (alternatively spliced molecules) using the HySeMaFi method, as are supported by the Miseq data (see arrow).

To effectively identify the alternatively spliced isoforms of genes, and confirm that they could be verified by different lines of evidence, we first performed PacBio long reads clustering analysis. Generally, when gene alleles and associated homologs were grouped against these results they typically shared the same alternative splicing patterns. The results of our clustering analysis showed that more than 80% of isoforms were grouped according to two types of molecule, but there were also more than 100 clusters that contained over 50 molecules (Fig. 5a). This result shows that varied isoforms, generated by a single gene, were widely found in our test samples. Based on our mapping methods and using the longest transcripts as reference data, we found that, in addition to the gene isoforms corresponding to the full complement of possible exons, at least 2,264 genes showed more than two alternative splice forms (isoforms). The majority of these genes corresponded to two-to-three isoforms, and this scenario is judged to be a reasonable representation of the true alternative splicing pattern in plants (Fig. 5b). Furthermore, we identified 498 genes that displayed at least three alternative splicing patterns (Supplementary Table S9), and in the majority of these cases the homologs of the genes have been previously reported to have alternative splicing patterns. In a parallel analysis, we used the Miseq data as the query set against which to align the longest and mapped contigs assembled in SGS, and the mapping results showed a high degree of consistency to the long reads data sets for the majority of genes analyzed (Fig. 5c and d). In addition, gene alleles sharing the same splicing pattern were also detected when the alignment was performed using BLAT with the similarity or identity level set below 100% (Fig. 5c,d).

### Gene expression defined by second sequencing using PacBio contigs as reference

**Figure 6:**
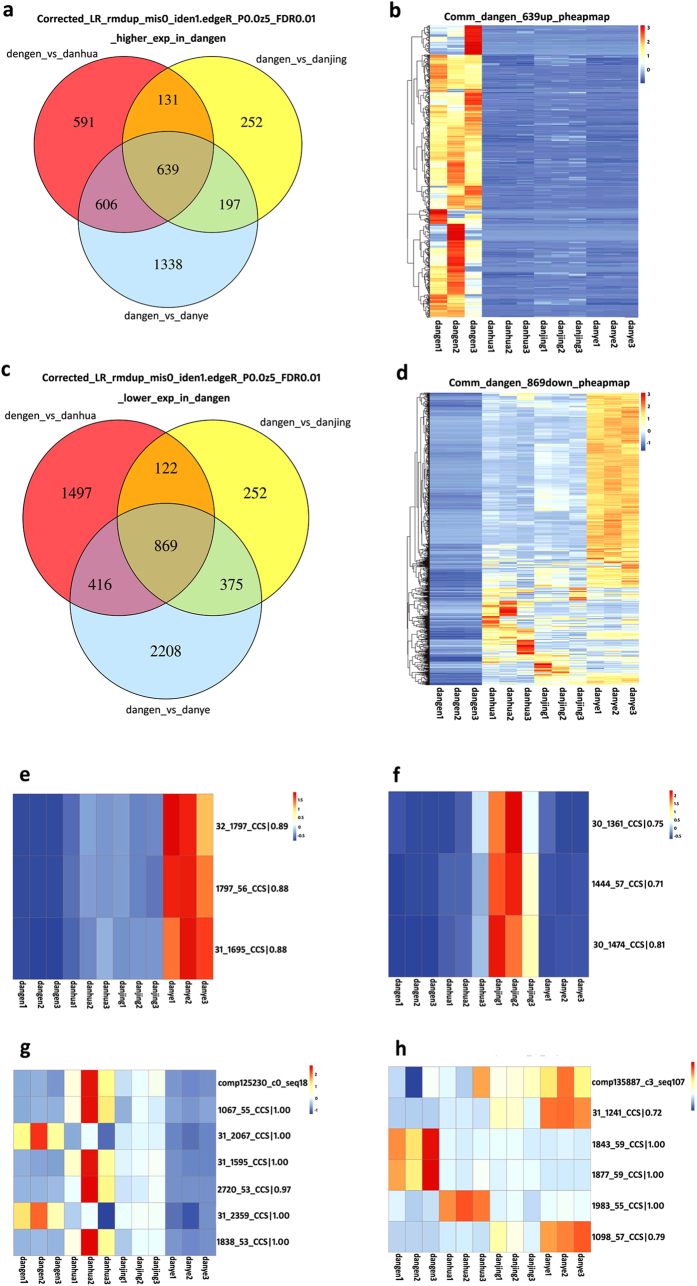
Characterization of the root, flower, stem and leaf transcriptome, and illustrating different expressions of genes specifically elevated or depressed in roots by TGS using hybrid sequencing and map finding. (**a**) The higher expression of isoforms specifically in roots; (**b**) Heat map shows the expression of 639 genes; (**c**) The lower expression of genes specifically in roots; (**d**) Heat map shows the expression of 869 genes; (**e,f**) The existing isoforms were detected to be differentially expressed in the tested samples, and the mapped contigs had been removed in the clustering analysis in the traditional RNA-Seq analysis; (**g,h**) The existing isoforms were detected to be differentially expressed in the tested samples and the mapped contigs had also been found to be differentially expressed.

To take account of the different expression levels of root-specific isoforms, the total number of 85,571 LSC corrected PacBio reads was used as the reference dataset. It was shown that 2,904, 1,618 and 3,868 individual isoforms had significantly higher expression levels in roots as compared to those in flower, stem and leaf tissues, respectively; of these, 639 transcript forms were consistently expressed most highly in roots (Fig. 6a). A heat map illustrates the expression of these 639 genes which was significantly (at least twofold) and consistently higher across triplicate root samples, as compared to triplicate samples of other tissues (Fig. 6b). On the other hand, 1,967, 1,219 and 2,780 isoforms had significantly lower expression levels in roots as compared to those in flower, stem and leaf tissues, respectively. Of these, 869 were consistently expressed at the lowest levels in roots, and this expression pattern was robustly supported for all of these genes by heat map analysis (Fig. 6c,d). In the traditional RNA-seq analysis which was based on the 490,981 total number of transcripts assembled in SGA, after the standard clustering analysis and removal of redundant sequences, 193,749 transcripts were finally used to make the reference dataset. According to this analysis, 896 transcripts were specifically expressed more highly in roots (Supplementary Fig. S6a) but a heat map shows that the significantly higher expression levels of these genes were not seen consistently across three different root samples (Supplementary Fig. S6b). In addition, 666 transcripts were found that were specifically expressed at lower levels in roots (Supplementary Fig. S6c,d). Comparison of the two analysis methods demonstrated that highly reproducible results, whether concerning up- or down-regulation, were obtained using our novel calculation methods and with corrected PacBio reads supplying the reference sequences. When comparing the mapping relationship between PacBio corrected long reads and contigs assembled from SGS, it should be considered that some transcripts would have been removed during the clustering steps, so the expression of mapped contigs may not correlate (Fig. 6e,f). If the same molecule is present in the PacBio corrected long reads and also in the contigs assembled from SGS, it should be presented with the same expression pattern in each analysis. True (Fig. 6g) or false molecules (Fig. 6h) in SGS were employed in the reference dataset in standard RNA-Seq analysis. Furthermore, abundance estimates for most of the highly-expressed isoforms from individual genes were entirely accurately predicted when taking the corrected PacBio reads as the reference dataset (Supplementary Fig. S6e,f).

## Discussion

### Hybrid sequencing and map finding involves a novel strategy to decipher gene splicing and expression

The hybrid sequencing and map finding strategy (HySeMaFi) described here is based on the following technical points. (1) SGS is employed and the assembly is conducted using low stringency settings for parameters such as Ker and coverage value. (2) SMRT sequencing is employed and the reads are corrected with short reads or other corrective methods. (3) A suitable method is used to establish the mapping relationship between assembled contigs and the PacBio corrected reads, and the longest molecules i.e. those containing all exons of a given gene are identified. (4) Using these longest molecules and PacBio corrected reads as input data, it is possible to determine alternative splicing patterns of genes by employing suitable alignment methods. (5) Using PacBio corrected reads as reference sequences against which to perform the RNA-seq analysis, it is possible to effectively illustrate the different expression patterns of various isoforms.

With regards to the traditional RNA-seq analysis, it should be noted that in order to get reliable information of a gene’s alternative splicing patterns, a contiguous genomic segment should be mapped, and there remains substantial difficulties with isoform identification and quantification^19^,^20^,^21^. Here, using the hybrid sequencing and map finding method we were able to obtain the longest contigs (i.e. those containing all exons/corresponding to the full-length gene without introns) by a similarity assembly approach to genome assembly. Therefore, using this method it is possible to perform corrected gene alternative splicing analysis, isoform identification and quantification analysis, and this approach may even facilitate greater accuracy in other downstream analyses. Our strategy takes advantage of the library constructions from outputs of short-read next-generation sequencing and achieves quantification of expression by calculating the mapped frequency of reads, whilst in addition taking advantage of the molecular completeness of the data from single molecule real-time (SMRT) sequencing (Fig. 1). Thus, the hybrid sequencing and map finding method involves a novel strategy to overcome the difficulties of *de novo* isoform discovery and can also remedy the insufficiencies of short-read sequencing with regard to accuracy of construction and quantification of isoforms^5^,^6^.

### Extensive mapping exists between the corrected PacBio read and contigs assembled from short reads

Theoretically, it is clear that various molecules, including both real and false forms, can be generated by employing different assembly methods based on short reads. Our study was able to verify this premise (Fig. 2) with the identification of the longest molecule for each gene, consisting of all exons, being assembled in combination with all other real as well as false molecules (Supplementary Fig. S2). In many cases, the longest of the short-reads assembled molecules is quite close to the full-length gene and has the highest similarity to the gene when compared to all of the isoforms derived from single molecule real-time sequencing. Thus, the longest molecules assembled from short reads have the potential to be used as reference sequences in subsequent analysis pathways when the reference genome is not available, as is the case for the majority of organisms. Through the use of suitable alignment methods, it is completely feasible that the longest molecules (i.e. those containing all of a gene’s exons), as assembled by short reads, could be used to map all of the real molecules (from the corrected PacBio reads) which contain all or some of the gene exons. Theoretically, the dataset of the longest molecules can play the role of a reference genome in mapping and also other subsequent analysis pathways (Fig. 1 and Supplementary Fig. S2). There are many available alignment methods, of which BLAT is commonly used to find regions in a corresponding genomic sequence which are similar to the query sequence and thus, determine the distribution of exonic and intronic regions of a gene. In our study, using the BLAT method and applying a high identity score, it was shown that extensive mapping existed between the corrected PacBio reads and the contigs assembled from short reads, although various corrective strategies were employed in the raw PacBio RS reads. In additional, the mapping method was tested and verified using the existing annotated isoform sets and the contigs datasets assembled from SGS in *Arabidopsis*.

### Alternative splicing patterns of genes are effectively detected by Hybrid sequencing and map finding

Alternative splicing is a mechanism by which multiple proteins can be produced from a single gene, and it is also a posttranscriptional mechanism that can regulate gene expression^22^. The corrected PacBio reads derived from single molecule real-time (SMRT) sequencing have the advantage that they contain all of the information originating from a single RNA molecule, and thus this data can be sufficient to detect gene splice sites^5^,^23^. Based on the genome data, NGS short-read data can identify splice sites by SpliceMap or Tophat, however, the largely incomplete and uncorrected assembled transcripts can substantially impede the direct identification of distinct isoforms. Thus, to date, there are no reports that document the successful use of NGS short-read data to identify splice sites in organisms as a way to over-come a short-fall in genome information. SMRT long-reads can be used as a way to detect isoforms effectively and the data has been employed to reveal the corresponding alternative splicing events in many organisms with an available reference genome^4^,^21^,^24^. To effectively decipher alternative splicing patterns in organisms lacking available genome information, we have developed the hybrid sequencing and map finding method and have demonstrated that it can directly identify distinct isoforms of individual genes (Fig. 5). Here, in accord with our initial hypothesis, the longest contigs assembled by short reads were found to be near to, or the same as the complete gene, providing the total isoform sequences covered the full genomic section corresponding to the gene (Supplementary Fig. S2). To verify the alternative splicing patterns detected by the hybrid sequencing and map finding technique, we aligned the MicroSeq data, which presented the actual sequence information at 400–500 bp lengths, to the longest contigs assembled by sequencing short reads. We found that almost all of the splicing sites detected were supported by the MicroSeq sequencing data (Fig. 5). Furthermore, we used the longest contigs that were detected to have at least four isoforms as the query sequences and conducted BLAT analysis of a non-redundant (NR) database. It was found in the majority of cases that the homologous genes were already reported to present comparable alternative splicing patterns (Supplementary Table S2). Thus, it is a robust finding that the events, such as exon skipping, intron retention and isoforms identification, are effectively detected by the hybrid sequencing and map finding method, especially in those deepen SGS and TGS cases. Certainly, it also present the limitation that the alternative splice borders are not well distinguished since it is short of those real intron information that is not transcribed.

### Tissue specific isoforms of genes were effectively deciphered by using the corrected PacBio reads as reference

In the calculations of isoform-specific gene expression based on SGS data, a short read consistent with 2 or more isoform types is usually regarded to be generated from the more abundant isoforms^25^. Isoform abundance estimates based on SGS data are highly sensitive to correction calculations and the true number of available isoform datasets^21^. In traditional RNA-Seq analysis, the two types of reference isoform are usually sourced from the existing libraries when the genome is available or, alternatively, from candidate isoform sets assembled by SGS analysis tools. RNA-seq analysis conducted using the existing isoform libraries as reference tends to increase the variability of the abundance estimates of the expressed isoforms since not all isoforms in the existing reference libraries are truly expressed in the tested sample, or alternatively, the reference library maybe incomplete. For RNA-seq using the isoform libraries assembled from short reads as reference, a candidate isoform set may include numerous false isoforms, so differing substantially from the true number of isoforms^21^. Thus, use of the error-corrected long reads is optimal for selecting corrected and true isoform datasets as the reference for a sample, and it will also enable much more reliable isoform quantification from SGS reads. In our study, we performed SMART and SGS sequencing for tissues of the roots, stems, leaves and flowers of *P.hybrida*, and used part of the SGS data to correct the PacBio long reads. These corrected PacBio long reads were then utilized as reference isoform libraries. In addition, we also performed traditional RNA-seq analysis with no reference sequence background. Our findings clearly showed that either elevated or depressed expression patterns in roots were both effectively detected using the corrected PacBio long reads as the reference isoform library (Fig. 6a,b). Furthermore, for the detected isoforms with an elevated root-specific expression, the majority of the homologous genes in specific species are known to also show high or low expression in root tissues (Supplementary Table S10 and Table S11). By contrast, use of the traditional RNA-Seq analysis method revealed that the longest assembled contigs mapping to the PacBio corrected reads may, or may not, truly be present in the final reference isoform library following clustering analysis of the assembled transcripts. Thus, it is inevitable that determining isoform expression levels using traditional RNA-Seq substantially increases the uncertainty of the abundance estimates of the expressed isoforms.

In summary, we have presented an optional strategy by which to extensively decipher gene splicing and expression by hybrid sequencing and map finding in organisms without a reference genome. In this method, mapping analysis is carried out between the corrected PacBio long reads and the contigs assembled from short reads (which correspond to all the isoforms of an individual gene), and the longest contig assembled from the short reads data is selected to group within the longest contig dataset. This latter dataset is used as the reference against which to detect the alternative splicing patterns of every gene by using reasonable alignment strategies. Additionally, however, by using redundancy-deducted PacBio long reads as established reference libraries, it will be possible to greatly increase the accuracy of the abundance estimates of the expressed isoforms.

## Materials and Methods

### Plant materials and sample preparation

*P. hybrida* plants were grown outside in the experimental field of Huazhong Agricultural University, Wuhan, China. Roots, stems, leaves and flowers of 9 plants were evenly harvested respectively for various sequencing library constructions. All samples were frozen in liquid nitrogen and stored at −80 °C until required for analysis.

### Hiseq and Miseq library construction and sequencing

Total RNA was isolated with TRIzol reagent (Invitrogen) and mRNA was purified using oligo (dT) magnetic beads (Dynabeads) according to the manufacturer’s instructions. Approximately 100–200ng of PolyA RNA was fragmented to perform first-strand cDNA using reverse transcriptase and random hexamer-primers. The second-strand cDNA was synthesized using DNA polymerase I and RNaseH. The cDNA was end-repaired and A-tailed, and Illumina paired-end adapters were added. After size selection on an agarose gel and PCR amplification, samples were sequenced on the Illumina Hi-Seq 2000 system, generating paired-end (PE) reads with a length of 2 × 100 bp. Samples were sequenced on the Illumina Mi-Seq system, generating paired-end (PE) reads with a length of 2 × 300 bp.

### PacBio library construction and sequencing

SMRTbell libraries were constructed by using the uniform mixed RNA form roots, stems, leaves and flowers and Pacific Biosciences’ 1.0 template prep kit (part 100-259-100) according to the manufacturer’s instructions. The synthesized cDNA was run on an agarose gel and 2 separate size ranges were fractionated: 1–2 kb, 2–3 kb. Each size fraction was extracted from the gel and treated according to Pacific Biosciences’ template preparation and sequencing protocol. The DNA/polymerase binding kit P5 (part 100-256-000) and v2 primers were used to make SMRTbell templates bound to polymerases. The polymerase–template complexes were bound to magbeads using Pacific Biosciences’ Magbead binding kit (part 100-133-600), and SMRT sequencing was then carried out on Pacific Biosciences’ real-time sequencer RTII by using DNA Sequencing Reagent 3.0 (part 100-254-800). All movie lengths were set to 240 min for each SMRT cell.

### Illumina sequencing and contigs obtained by *de novo* assembly

For the SGS sequencing short reads data(Hiseq) in *Arabidopsis* (SRR2898686, SRR2898687 and SRR2898688) were downloaded. With those the sequencing raw datasets from *P.hybrida*, we carried out a stringent filtering process of raw sequencing reads before the transcriptome assembly. Both raw reads of Miseq and Hiseq were cleaned by removing adapter sequences, non-coding RNA (such as rRNA, tRNA and miRNA), and low-quality sequences (reads with ambiguous bases ‘N’). Hiseq clean reads were assembled with Trinity^26^ using the following parameters: –edge-thr = 0, –flow-thr = 0 and the remainder default parameters. This meant keeping the edge as much as possible in Butterfly. Miseq clean reads were overlapped by FLASH-1.2.6 using the following parameters: -M 200 -r 300 -f 500 -s 50.

### Full-length corrected transcripts collection in SMART sequencing

Full-length transcripts which contained poly-A tails, 5′ primers and 3′ primers were obtained by using Pacific Biosciences’ SMRT analysis software (v2.3.0). Any reads shorter than 200 bp and those with a predicted accuracy lower than 75 were removed. The accuracy of full-length transcripts was generally not as high as SGS short reads. LSC 0.3.1^9^ was used to correct full-length transcripts. The options “I_nonredundant” was set to “N” and “I_RemoveBothTails” was set to “Y”. The length of pseudo chromosomes was 50,000,000 and the length of sequence gap between long reads was 100. Minimum number of non’N’ after compressing was set to 39, and “maximum ‘N’ allowed after compressing” was set as 1.

### Mapping finding between corrected PacBio reads and contigs and best-hit longest contigs selection

In our hybrid sequencing and map finding method, the common BLAT alignment algorithm was used, the following hypothesis was proposed, and we formulated six likelihood functions:


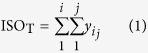



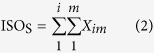



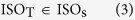



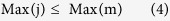



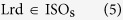



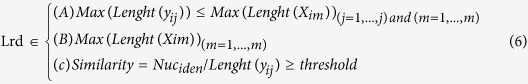


where Iso_T_ is the isoform library defined from the SMRT sequencing, Iso_S_ is the contigs (isoforms) library assembled from short reads based on the Illumina sequencing. The two types of isoforms libraries represent the isoforms sets of all the expressed genes in the specific cell, tissue or organ, as defined by two different sequencing methods. y_ij_ indicates the jth isoform of the ith gene in the isoform library derived from SMRT sequencing; X_im_ indicates the mth isoform of the ith gene assembled from short reads. Function (4) shows that the number of true expressing isoforms of each gene obtained from SMRT sequencing is less than that of the theoretical assembly from short reads. Lrd represents the set of longest contigs that are selected from the contigs (isoforms) library assembled from short reads (shown in function (5)) and the members of L_rd_ should meet the demands listed in function (6). As shown in function (6), firstly, the length of the mapping isoform of an individual gene in Iso_S_ should be equal or longer than those corresponding isoforms derived from SMART sequencing (function A), and the longest one represents the form that contains almost all full-length exons of that gene (function B). Secondly, those mapping molecules should have a high level of similarity (we suggest typically >99% threshold), that is the rate of the identified nucleotides (Nuc_iden_) to the length of the mapped isoform (Length(y_ij_)) in SMRT sequencing (function B). Based on the theoretical hypothesis, we developed a script for performing the map finding process to identify the longest contigs datasets. In addition, the method developed by us was also used to carry out the redundancy removal analysis in our PacBio corrected long reads as described here. Thus, if the length of read A was longer than read B, and in addition read B did not have overhangs, the similarity was higher than 0.99 and there were no gaps between the two reads, then we concluded that read B was a duplication of read A, and so deleted the B reads. By aligning the duplication-removed and corrected long reads (DRCLR) to contigs assembled by Trinity with a 99% threshold, we assigned the longest contigs identified to be DRCLR, regardless of any gaps in the reference contigs.

### Alternative splicing analysis

Based on our mapping strategy, after BLAT alignment software was used to align DRCLR to the contigs assembled by short reads from SGS (Illumina Hi-Seq) by Trinity using default parameters, the longest contigs, representing 99% of our defined similarity, were selected to make up the reference library. We defined the alignment gaps longer than 50 bp as splices. Different lengths or sites of the gaps were defined as alternative splicing.

### Isoform differential expression analysis

Isoform expression levels among various samples were identified based on the short reads datasets and using the isoforms libraries, yielded from the SMRT sequencing analysis and from the contigs assembly followed by clustering as *per* traditional RNA-Seq, as reference sequences. Extracts of four different organs (root, stem, leaf and flower) were used as examples of the analysis in this study. The expression analysis from Illumina reads of different tissues was carried out with bowtie (v1.1.1) and rsem (v1.2.9)^27^ using default parameters. P value < 0.05, FDR < 0.01, and a fold change equal or greater than 2-fold were used as the screening cutoffs for determining extremely significant differential gene expression between two samples. Highly expressed isoforms with root-specific characteristics were used as examples to test the effectiveness of the two parallel abundance estimates for isoform analysis.

## Additional Information

**How to cite this article**: Ning, G. *et al*. Hybrid sequencing and map finding (HySeMaFi): optional strategies for extensively deciphering gene splicing and expression in organisms without reference genome. *Sci. Rep.* **7**, 43793; doi: 10.1038/srep43793 (2017).

**Publisher's note:** Springer Nature remains neutral with regard to jurisdictional claims in published maps and institutional affiliations.

## Supplementary Material

Supplementary Information

Supplementary Dataset 1

Supplementary Dataset 2

Supplementary Dataset 3
