# Supplementary material for: Hybrid sequencing and map finding (HySeMaFi): optional strategies for extensively deciphering gene splicing and expression in organisms without reference genome
Source: Sci Rep. 2017 Mar 8;7:43793. doi: 10.1038/srep43793 (PMC5341560; doi:10.1038/srep43793)
Supplement: Supplementary Information [file srep43793-s1.pdf]

Hybrid sequencing and map finding (HySeMaFi): optional strategies for extensively deciphering gene splicing and expression in organisms without reference genome

Guogui Ning*[1], Xu Cheng[2], Ping Luo[1], Fan Liang[3], Zhen Wang[1], Guoliang Yu[1], Xin Li[1], Depeng, Wang[3], Manzhu Bao*[1]

Address:

[1]Key laboratory of Horticultural Plant Biology, Ministry of Education,

College of Horticulture and Forestry Sciences,

Huazhong Agricultural University, Wuhan, P. R. China

[2]Medical Research Institute, School of Medicine, Wuhan University, Wuhan, P. R. China

[3]Nextomics Biosciences Co., Ltd., Wuhan, Hubei, China

*Author for correspondence: **Guogui Ning & Manzhu Bao**

E-mail: **ggning@mail.hzau.edu.cn; mzbao@mail.hzau.edu.cn**

Fax: +86 27 8728 2010

Phone: +86 27 8728 6928

supplementary information

Figure S1. Strategy to decipher the genes splicing and expression in the transcriptome of non model organism.

Figure S2. Schedule to the mapping strategy between PacBio corrected long reads (Left) and contigs assembled from SGS (Right) based on isoforms from one theoretical gene.

Figure S3. Distribution of extended fragments length in Miseq analysis.

Figure S4. Number distribution at varied Read of insert Length and Quality respectively.

Figure S5. Number distribution at varied Read Length of Full-length Non-Chimeric Reads.

Figure S6. Characterizing the root, flower, stem and leaf transcriptome and illustrating different expressing genes specific or not specific to root by short reads using traditional RNA-Seq methods. (A) The higher expression genes specific to roots; （B）Heap map shows the expression of 896 genes; （C）The lower expression genes specific to roots; （D）Heap map shows the expression of 666 genes; （E-F）Expression analysis to the mapping derived transcripts between SGS and TGS. Note: Red high expression, blue low expression, left cluster gene tree.

Table S1. Summary of sample short reads after clearing in SGS sequencing from Petunia.

Table S2. Summary of transcripts assembled from short reads data by trinity with different parameters from Petunia.

Table S3. Summary of sample Miseq reads from Miseq sequencing from Petunia.

Table S4. Summary of processed sample Miseq reads from Miseq sequencing from Petunia.

Table S5. Summary of sample short reads after clearing in SGS sequencing from Arabidopsis.

Table S6. Summary of transcripts assembled from short reads data by trinity with different parameters from Arabidopsis.

Table S7. Summary of sample reads of insert in SMRT sequencing from Petunia.

Table S8. Classify of reads of insert in SMART sequencing from Petunia.

Table S9. The identified genes that displayed at least three alternative splicing patterns.

Table S10. The majority of genes, with the homologous genes in specific species, show high expression in root determined by using the corrected PacBio long reads as the reference.

Table S11. The majority of genes, with the homologous genes in specific species, low expression in root determined by using the corrected PacBio long reads as the reference.

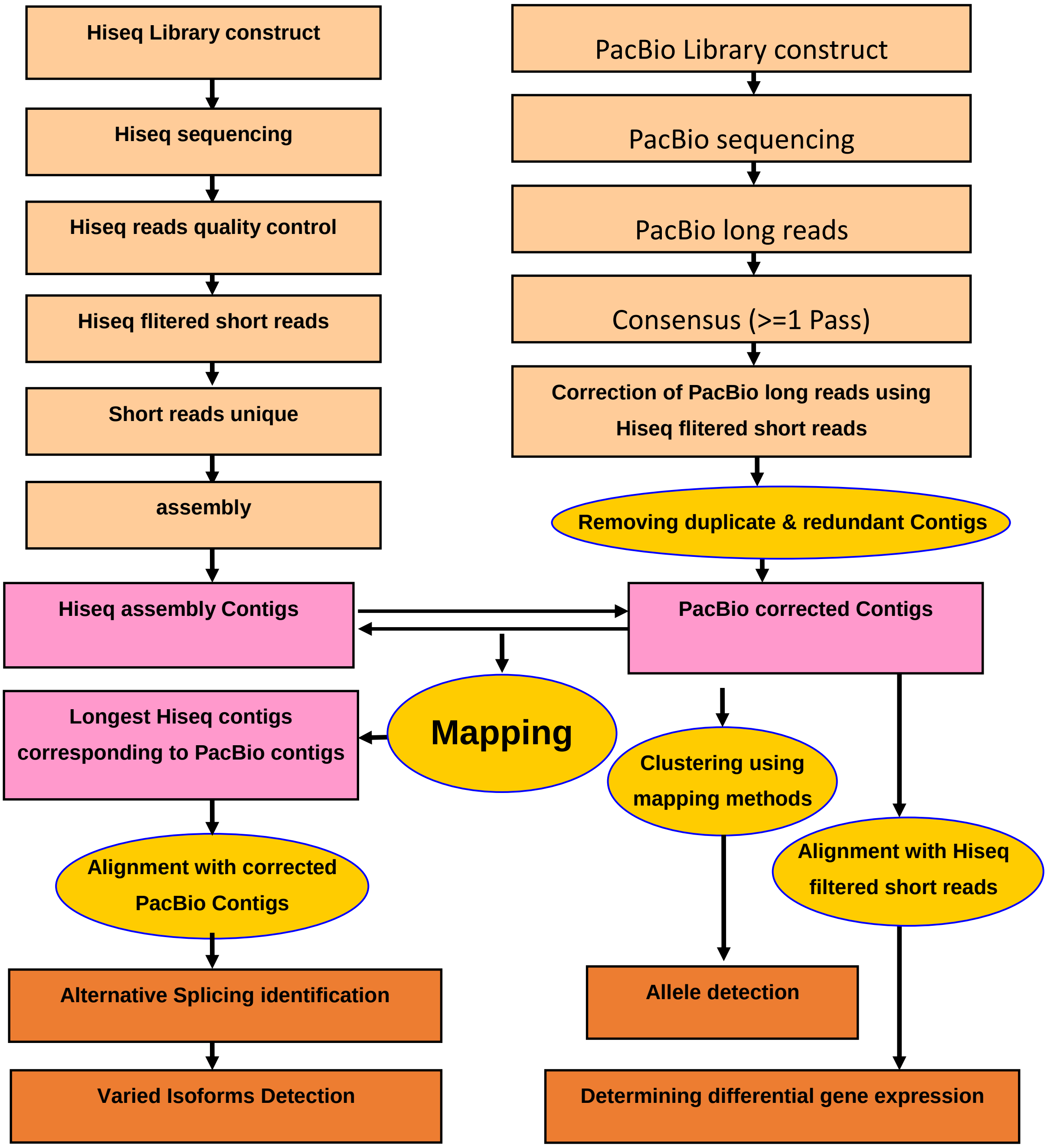

Figure S1. Strategy to decipher the genes splicing and expression in the transcriptome of non model organism.

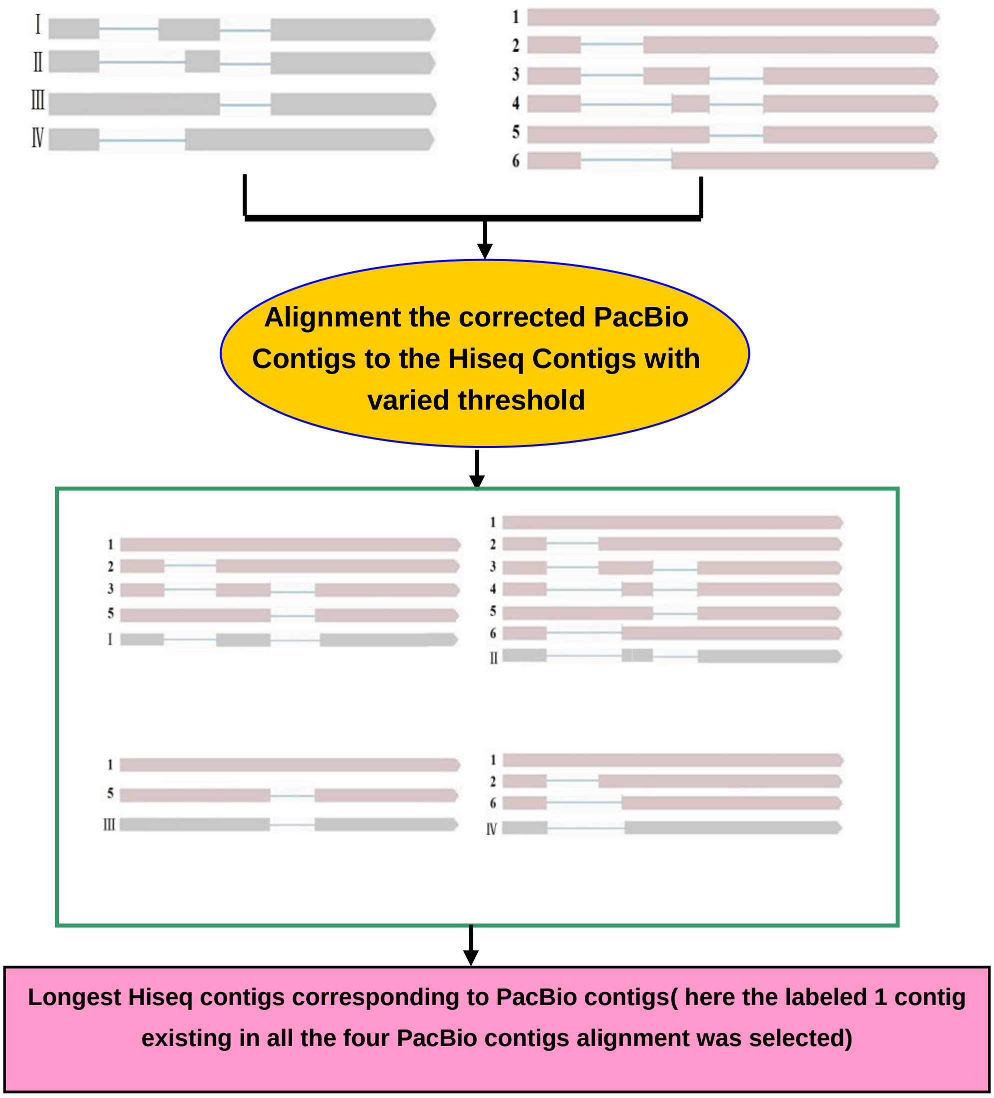

Figure S2. Schedule to the mapping strategy between PacBio corrected long reads (Left) and contigs assembled from SGS (Right) based on isoforms from one theoretical gene.

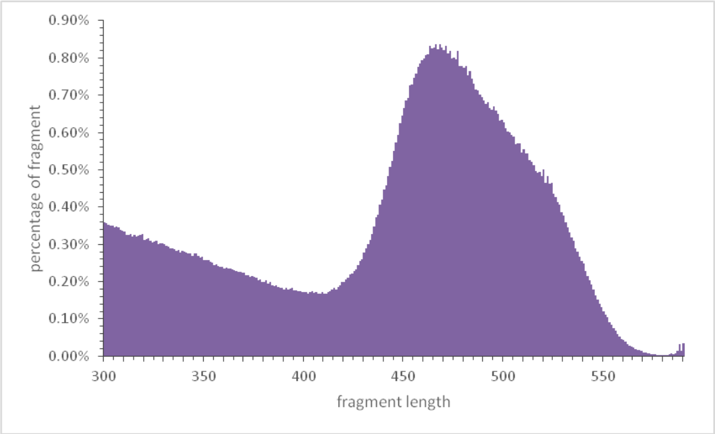

Figure S3. Distribution of extended fragments length in Miseq analysis.

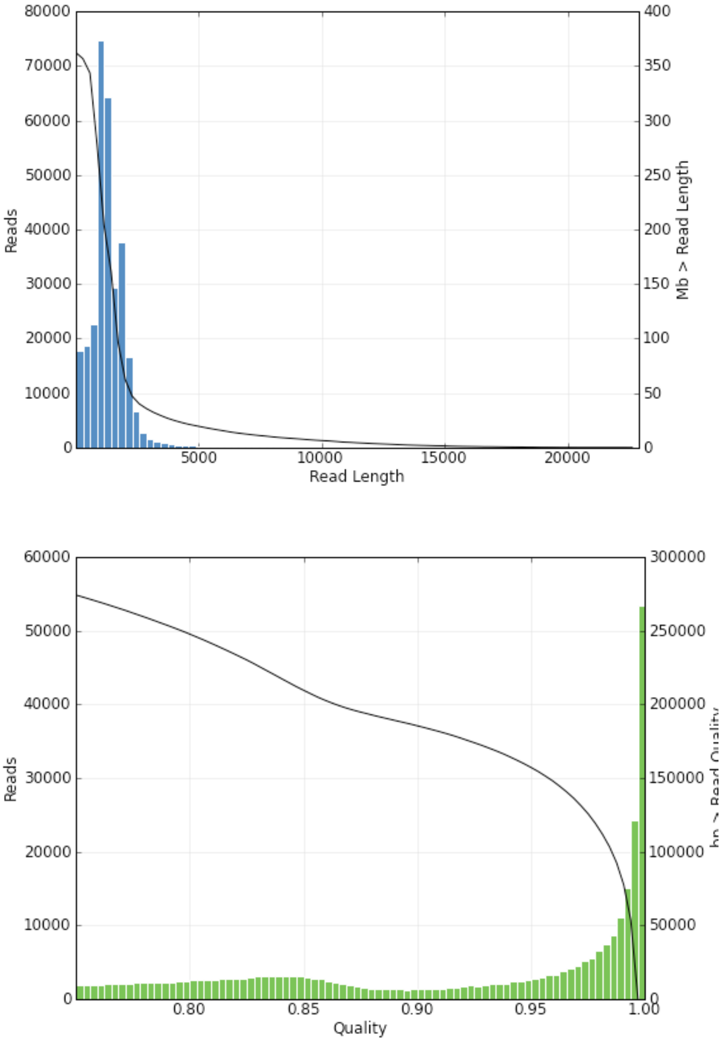

Figure S4. Number distribution at varied Read of insert Length and Quality respectively.

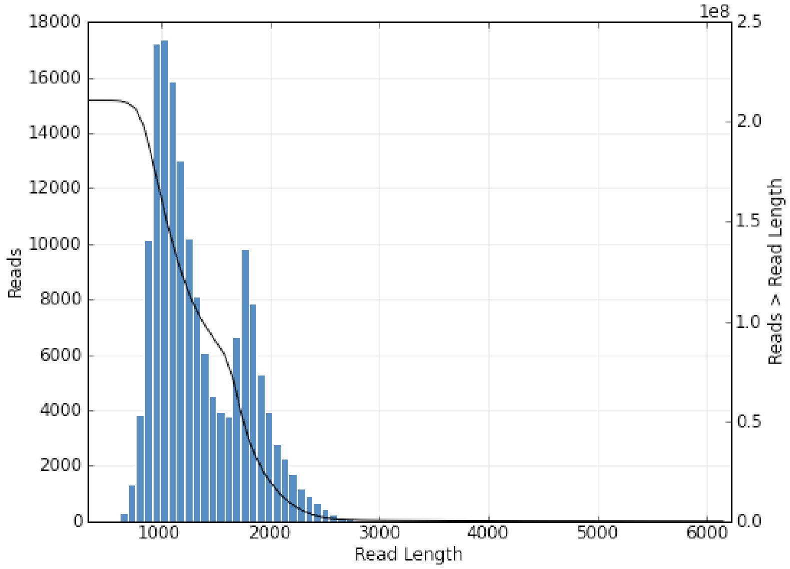

Figure S5. Number distribution at varied Read Length of Full-length Non-Chimeric Reads.

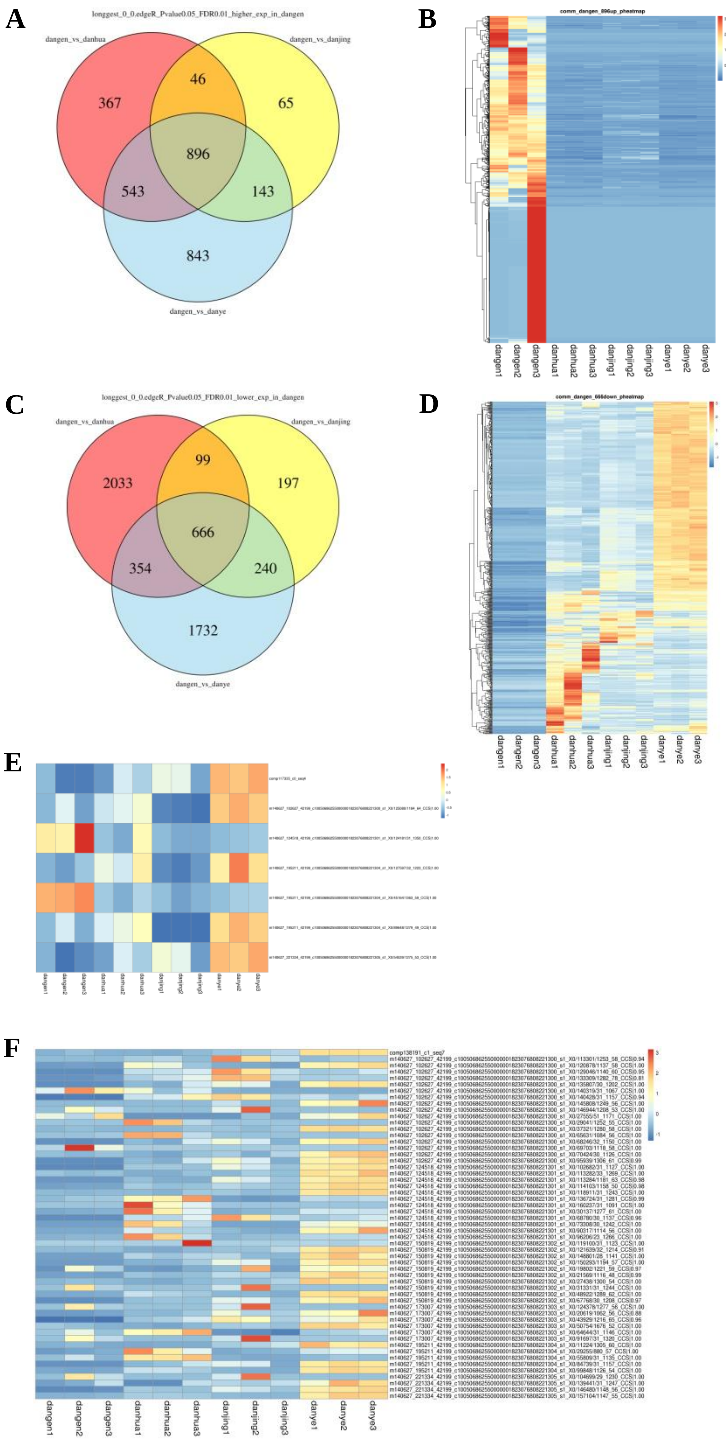

Figure S6. Characterizing the root, flower, stem and leaf transcriptome and illustrating different expressing genes specific or not specific to root by short reads using traditional RNA-Seq methods. (A) The higher expression genes specific to roots; （B）Heap map shows the expression of 896 genes; （C）The lower expression genes specific to roots; （D）Heap map shows the expression of 666 genes; （E-F）Expression analysis to the mapping derived transcripts between SGS and TGS. Note: Red high expression, blue low expression, left cluster gene tree.

Table S1. Summary of sample short reads after clearing in SGS sequencing from Petunia.

Sample	Read Length	Clean reads	Clean Bases	Q20 Rate(%)	Q30 Rate(%)
dangen1	100	83,536,154	8,353,615,400	0.95	0.90
dangen2	100	79,683,542	7,968,354,200	0.95	0.90
dangen3	100	63,482,438	6,348,243,800	0.95	0.90
danhua1	100	80,752,172	8,075,217,200	0.95	0.90
danhua2	100	81,043,876	8,104,387,600	0.95	0.90
danhua3	100	87,797,476	8,779,747,600	0.95	0.90
danjing1	100	74,661,844	7,466,184,400	0.94	0.89
danjing2	100	74,147,972	7,414,797,200	0.95	0.90
danjing3	100	81,139,600	8,113,960,000	0.95	0.90
danye1	100	78,068,476	7,806,847,600	0.95	0.90
danye2	100	81,918,232	8,191,823,200	0.95	0.90
danye3	100	79,269,758	7,926,975,800	0.95	0.90

Table S2. Summary of transcripts assembled from short reads data by trinity with different parameters from Petunia.

Sample	Total length	Total genes	Average length	N50 length	Max length	Min length
**Trinity lower para**	809,209,091	490,981	1,648.15	2,930	37,250	201
**Trinity default para**	575,798,199	412,941	1,394.38	2,579	19,688	201
**Trinity lower para**	337,981,378	301,386	1,121.42	2,203	37,250	201

Table S3. Summary of sample Miseq reads from Miseq sequencing from Petunia.

Reads	Length	# of reads	# of reads with N	# of bases	# of HQ bases	HQ bases rate(%)
Raw R1	300	10,354,752	826	3,106,425,600	2,943,860,296	94.77
Raw R2	300	10,354,752	249	3,106,425,600	2,793,606,115	89.93
Clean R1	300	10,076,596	474	3,022,978,800	2,879,895,198	95.27
Clean R2	300	10,076,596	31	3,022,978,800	2,735,718,133	90.50

Table S4. Summary of processed sample Miseq reads from Miseq sequencing from Petunia.

type	# of Reads	BaseNumber	Average Length	Average Q	Q20
R1 reads	5,758,294	1,727,488,200	300	64.7	0.80
R2 reads	5,758,294	1,727,488,200	300	62.19	0.73
Extended fragments	4,596,458	2,041,011,806	444	69.24	0.97

Table S5. Summary of sample short reads after clearing in SGS sequencing from Arabidopsis.

Sample	Clean Read Number	Clean Base Number	Q30 Rate
SRR2898686	36,146,222	3,650,296,892	95.69%
SRR2898687	40,746,426	4,114,983,071	95.79%
SRR2898688	38,886,698	3,927,152,800	95.65%

Table S6. Summary of transcripts assembled from short reads data by trinity with different parameters from Arabidopsis.

Sample	Total length	Total genes	Average length	N50 length	Max length	Min length
Trinity lower para	55,673,892	44,934	1,239	2,007	16,568	201
Trinity default para	35,516,195	44,914	791	1,121	8,166	200

Table S7. Summary of sample reads of insert in SMRT sequencing from Petunia.

Sample	Reads Of Insert	Read Bases Of Insert	Mean Read Length Of Insert	Mean Read Quality Of Insert	Mean Number Of Passes
A01_1	51,204	58,620,034	1,144	0.9111	5.87
A01_2	49,341	57,266,291	1,160	0.9318	7.36
A01_3	46,767	53,633,722	1,146	0.9307	7.33
B01_1	49,107	75,009,056	1,527	0.9179	6.00
B01_2	49,707	76,344,222	1,535	0.9178	5.99
B01_3	53,416	84,288,879	1,577	0.9243	6.32

Table S8. Classify of reads of insert in SMART sequencing from Petunia.

	Value	Rate
# of reads of insert	299,542	-
# of five primer reads	188,001	62.76%
# of three primer reads	201,966	67.43%
# of poly-A reads	201,593	67.30%
# of filtered short reads	18,821	6.28%
# of non-full-length reads	119,993	40.06%
# of full-length reads	160,728	53.66%
# of full-length non-chimeric reads	160,293	53.51%
# of chimeric reads	435	0.15%
Average full-length non-chimeric read length	1,350	0.45%
